# A caffeic acid-loaded hydrogel attenuates inflammation and accelerates infected wound healing

**DOI:** 10.3389/fbioe.2026.1851700

**Published:** 2026-06-10

**Authors:** Mengwei Zhang, Ye Zhang, Hanxi Luo, Li bing Sun, Ting Li, Yifan Chen, Bo Chen, Ming Li, Tingmin Xu

**Affiliations:** 1 Trauma Center, Peking University People’s Hospital; Key Laboratory of Trauma Treatment and Neural Regeneration (Peking University) Ministry of Education, National Center for Trauma Medicine, Beijing, China; 2 Graduate School, Hebei North University, Zhangjiakou, Hebei, China; 3 School of Basic Medical Sciences, Peking University Health Science Center, Beijing, China

**Keywords:** antioxidant activity, caffeic acid, double-network hydrogel, immune regulation, infected wounds

## Abstract

**Introduction:**

Infected wounds are often accompanied by persistent bacterial colonization, dysregulated inflammation, and aggravated oxidative stress, which worsen the local microenvironment and hinder tissue repair. Therefore, the development of bioactive dressings with antibacterial, antioxidant, and inflammation-regulating properties is of great importance. This study aimed to construct a caffeic acid-loaded double-network hydrogel and evaluate its potential for the treatment of infected wounds.

**Methods:**

The porous structure, fluid absorption capacity, and release behavior of the hydrogel were characterized. Its cytocompatibility, hemocompatibility, antibacterial activity, antioxidant capacity, and regulatory effects on macrophages and fibroblasts were further evaluated *in vitro*. In addition, an infected full-thickness skin defect model was established to assess its therapeutic efficacy *in vivo*.

**Results:**

The hydrogel exhibited a favorable porous structure and fluid absorption capacity, and achieved sustained release of caffeic acid. It also showed good cytocompatibility and hemocompatibility, along with strong inhibitory effects against *Staphylococcus aureus* and *Escherichia coli*, and enhanced free radical scavenging activity. In addition, the hydrogel upregulated Arg-1 expression and downregulated iNOS expression in macrophages, promoting macrophage polarization toward the anti-inflammatory M2 phenotype, while also enhancing the expression of Col I and Col III in fibroblasts. *In vivo*, the hydrogel improved local infection status, accelerated wound healing, and enhanced re-epithelialization and collagen deposition.

**Conclusion:**

These findings indicate that the caffeic acid-loaded double-network hydrogel not only reduces bacterial burden and oxidative stress in infected wounds, but also improves the local reparative microenvironment by regulating inflammation-related cell phenotypes and promoting extracellular matrix synthesis, thereby accelerating tissue regeneration. This study provides a new strategy for the design of bioactive dressings for infected wounds and also offers experimental evidence for local therapeutic approaches that integrate infection control with tissue repair.

## 1 Introduction

Wound repair is a major challenge in clinical medicine and encompasses a wide range of wound types, including burns, diabetic ulcers, and infected wounds (Wang W. et al., 2023). As a complex and highly coordinated biological process, wound healing involves several overlapping phases, including hemostasis, inflammation, proliferation, and remodeling. However, bacterial infection, excessive inflammatory responses, and oxidative stress can disturb this tightly controlled cascade, thereby resulting in delayed healing or even wound repair failure (Yue et al., 2023). Wound infection imposes a substantial clinical burden. A study published in 2017 showed that surgical site infection (SSI) is among the most frequently reported types of hospital-acquired infection (HAI), and in European hospitals, the economic burden associated with patients who develop SSI is approximately twice that of patients without SSI (Badia et al., 2017). Although antibiotics and nanomaterial-based antimicrobial agents are widely used in clinical practice, the emergence of antibiotic resistance caused by antibiotic overuse, together with the cytotoxicity and environmental risks associated with nanoparticles such as silver, zinc, and copper, has limited their long-term application (Dong et al., 2022; Meng et al., 2024). Accordingly, recent biomaterial strategies increasingly emphasize simultaneous infection control and microenvironment regulation. For example, a chitosan-Prussian blue nanozyme system integrated antibacterial, antioxidant, and anti-inflammatory effects, supporting the value of multifunctional non-antibiotic approaches for bacteria-infected wounds (Wang et al., 2024). In addition, excessive inflammatory infiltration can interrupt the healing process (Li M. et al., 2025). Studies have shown that dysfunction of immunoregulatory cells, including macrophages, and fibroblasts, contributes to persistent inflammation and impaired tissue regeneration (He et al., 2024). Notably, excessive reactive oxygen species (ROS) are recognized as one of the major mechanisms responsible for delayed healing in infected wounds. While physiological levels of ROS contribute to normal wound repair at the early stage through antimicrobial activity and signaling regulation, sustained ROS overproduction induces oxidative stress, causing oxidative damage to lipids, proteins, and DNA, promoting local cell apoptosis, and impairing the normal functions of fibroblasts, keratinocytes, and endothelial cells (Hunt et al., 2024). Meanwhile, excessive ROS accumulation further aggravates inflammation, hinders angiogenesis and extracellular matrix remodeling, and causes the wound to remain in a prolonged inflammatory state, ultimately resulting in impaired tissue repair and chronicity (Hunt et al., 2024; Li et al., 2022; Wang G. et al., 2023).

Caffeic acid (3,4-dihydroxycinnamic acid, CA) is an important phenolic compound naturally derived from plants such as coffee beans, and its molecular structure contains both phenolic and acrylic active functional groups (Benbettaieb et al., 2018). The use of plant-derived bioactive compounds in wound repair is further supported by recent evidence that Hypericum aqueous extract promotes skin wound healing through PI3K/Akt-related regulation (Xiong et al., 2025). Previous studies have demonstrated that caffeic acid possesses a broad range of biological and pharmacological activities, including antibacterial, anti-inflammatory, antidiabetic, anticancer, antithrombotic, and antihypertensive effects (Nam et al., 2020; Stasilowicz-Krzemien et al., 2023; Zhou et al., 2023; Khan et al., 2021). Owing to its polyphenolic structure, which enables free-radical scavenging through hydrogen atom transfer and single-electron transfer mechanisms, caffeic acid exhibits potent antioxidant activity and has therefore been widely investigated for the treatment of infectious diseases mediated by pathogens such as bacteria, fungi, and viruses (Pandey and Rizvi, 2009). With respect to its antibacterial activity, studies have shown that caffeic acid can interact with the bacterial cell wall and cytoplasmic membrane and subsequently enter the cell to exert biological effects (Andrade et al., 2015). Its antibacterial action is primarily attributed to increased membrane permeability, leakage of intracellular components, interference with enzyme activity, and disruption of nucleic acid homeostasis, thereby leading to bacterial physiological dysfunction and death (Park and Kang, 2021; Kepa et al., 2018). Beyond its antibacterial activity, caffeic acid has also been widely recognized for its anti-inflammatory potential. Previous studies have shown that it can suppress inflammatory mediator production and downregulate pro-inflammatory molecules in activated macrophages (Yang et al., 2013). However, its biomedical application is still limited by poor water solubility, low stability, and restricted bioavailability (Stanciauskaite et al., 2023; Wang C. et al., 2023). Therefore, developing suitable carrier systems to improve its local delivery and biological efficacy has become an important strategy for expanding its use in wound repair (Zhang et al., 2024; Tang Y. et al., 2024). Similar carrier-based design has also been used for other natural small molecules, such as baicalein-loaded antibacterial hydrogels that modulate macrophage-associated skin repair responses (Shang et al., 2025).

To promote tissue repair, a variety of wound dressings have been developed. An ideal wound dressing should possess excellent biocompatibility, moisture retention, air permeability, antibacterial activity, and antioxidant capacity, while also being able to absorb wound exudate and facilitate healing (Ma et al., 2024). Traditional dressings have shown certain therapeutic benefits; however, their relatively simple network structures and dry properties limit their broader application (Tang Z. et al., 2024; Ye et al., 2024). In contrast, hydrogels, owing to their high water content and three-dimensional network architecture, can provide a moist environment for wounds and promote the exchange of oxygen and carbon dioxide, thereby accelerating healing (Wang et al., 2022). However, conventional hydrogel dressings often exhibit limited functionality and are insufficient to meet the multiple demands of healing complex infected wounds. Through the synergistic integration of dynamic covalent bonds and physical crosslinking, double-network hydrogels not only display excellent mechanical properties and self-healing capability, but also enable sustained and controlled drug release (He et al., 2021). Recent injectable hydrogel systems further support the general design principle that hydrogel networks can organize bioactive components for localized release and microenvironment regulation (Ouyang et al., 2026). Chitosan, due to its positively charged nature, can bind to negatively charged bacterial membranes and disrupt bacterial structures, thereby exhibiting remarkable antibacterial activity (Zhao et al., 2023). Its derivative, carboxymethyl chitosan (CMCS), not only retains the biocompatibility and antibacterial activity of chitosan, but also exhibits good water solubility, making it widely applicable in the field of wound dressings (Li M. et al., 2025).

**FIGURE 1:**
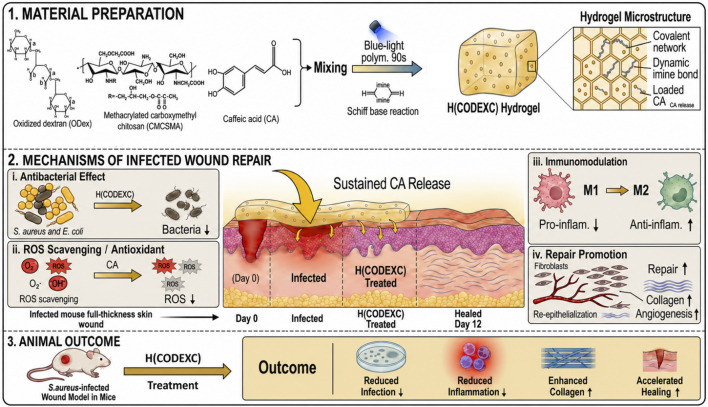
Graphic Abstract. Schematic illustration of the construction of a caffeic acid-loaded double-network hydrogel and its therapeutic effects on infected wound healing.

Double-network hydrogels, through the synergistic effect of a stable covalent network and a dynamic reversible network, combine favorable mechanical properties, self-healing ability, and sustained drug-release characteristics (Zhan et al., 2024). In this study, we constructed a CMCSMA/ODex-based double-network hydrogel and further incorporated caffeic acid to integrate the structural support of the hydrogel with the antibacterial, antioxidant, and inflammation-regulating activities of caffeic acid. Owing to the good biocompatibility, water solubility, and intrinsic antibacterial activity of carboxymethyl chitosan, this system is well suited for wound dressing applications. We therefore hypothesized that this strategy could improve the dysregulated microenvironment of infected wounds and promote tissue repair (Figure 1).

## 2 Materials and methods

### 2.1 Materials

Carboxymethyl chitosan (CMCS; Mw = 240 kDa; degree of substitution = 90%), dextran, methacrylic anhydride (MA, 94%), sodium periodate, and hydroxylamine hydrochloride were purchased from Shanghai Macklin Biochemical Co., Ltd (Shanghai, China). Lithium phenyl(2,4,6-trimethylbenzoyl)phosphinate (LAP, 98%) was obtained from Suzhou Yongqinquan Intelligent Equipment Co., Ltd (Jiangsu, China). All other reagents were purchased from Sinopharm Chemical Reagent Co., Ltd (Shanghai, China).

### 2.2 Synthesis of CMCSMA and ODex

CMCSMA was synthesized by dissolving 2 g of CMCS powder in 200 mL of deionized water. After complete dissolution, 2 mL of MA was added dropwise. The reaction was conducted in an ice-water bath at pH 8 for 8 h, followed by dialysis and lyophilization. ODex was synthesized by dissolving 2 g of dextran in 20 mL of deionized water, followed by the addition of 1.08 g NaIO_4_ (2.7% g/mL, 40 mL). The reaction proceeded in the dark for 4 h. Subsequently, 1 mL of ethylene glycol solution was added dropwise, and the mixture was stirred for 15 min to terminate the reaction. The product was dialyzed using a dialysis membrane (MWCO 3500) and then freeze-dried.

### 2.3 Preparation of hydrogels

First, a 5 wt% CMCSMA solution containing LAP (0.25% w/v relative to the CMCSMA content) and a 4 wt% ODex solution were prepared separately. A pure CMCSMA hydrogel was prepared by mixing the CMCSMA precursor with an equal volume of water, followed by blue-light irradiation for 90s; this hydrogel was denoted as H(CMCSMA). Using the same procedure, a bicomponent hydrogel was prepared by mixing the CMCSMA precursor with the ODex precursor and was denoted as H(CODEX). Subsequently, 5 mg of caffeic acid (CA) was added to 1 g of the CODEX precursor to obtain a drug-loaded hydrogel, denoted as H(CODEXC).

### 2.4 Characterization of hydrogels

Swelling test: Freeze-dried samples with identical masses were weighed and recorded as $W_{0}$ , and then immersed in phosphate-buffered saline (PBS, pH 7). At predetermined time intervals, the hydrogels were weighed until equilibrium was reached. The equilibrium swelling ratio was calculated using the following equation:

$$\text{Swelling ratio }\left(\%\right)=\frac{W_{t}-W_{0}}{W_{0}}\times 100\%$$

where $W_{0}$ represents the initial weight of the hydrogel and $W_{t}$ represents the weight of the hydrogel at a given time point.

Degradation test: The prepared hydrogels were freeze-dried for 48 h and weighed to obtain the initial dry weight ( $W_{0}$ ). The freeze-dried hydrogels were then immersed in PBS (pH 7) and incubated in a constant-temperature shaker at 37 °C and 200 rpm. At predetermined time points, the hydrogels were removed, washed two to three times with deionized water, freeze-dried again for 48 h, and weighed to obtain $W_{t}$ . The degradation ratio was calculated using the following equation:

$$\text{Degradation ratio }\left(\%\right)=\frac{W_{0}-W_{t}}{W_{0}}\times 100\%$$

where $W_{0}$ is the initial dry weight and $W_{t\;}$ is the dry weight at time t .

### 2.5 *In Vitro* release study of CA

The *in vitro* cumulative release of CA was measured using a UV spectrophotometer (TU-1810, Beijing Puxi General Instrument Co., Ltd., Beijing, China). Briefly, a series of CA standard solutions were prepared, and their absorbance at 243 nm was measured to construct a standard calibration curve. The hydrogel was then immersed in 5 mL of phosphate buffer at 37 °C. At predetermined time points (0, 2, 4, 8, 10, 12, 24, 48 and 72 h), 1 mL of release medium was withdrawn and replaced with an equal volume of fresh phosphate buffer to maintain the original volume. The absorbance of the collected solution was then measured. The cumulative release rate of CA was calculated as follows:

$$\text{Cumulative release rate }\left(\%\right)=\frac{R_{t}}{R_{i}}\times 100\%$$

where $R_{t}$ represents the total amount of drug released at time t , and $R_{i}$ represents the total amount of CA loaded in the hydrogel.

### 2.6 ^1^H NMR characterization

The chemical structures of DEX, ODex, CMCS, and CMCSMA were characterized by proton nuclear magnetic resonance (^1^H NMR) spectroscopy. Briefly, DEX, ODex, CMCS, and CMCSMA were dissolved in D_2_O, and the 1H NMR spectra were recorded at room temperature using an NMR spectrometer.

### 2.7 FTIR characterization

Fourier-transform infrared spectroscopy (FTIR) was used to further analyze the chemical structures of DEX, ODex, CMCS, CMCSMA, CA, H(CODEX), and H(CODEXC). Freeze-dried samples were ground into powders before measurement. FTIR spectra were collected over the range of 4,000–400 cm^-1^ with being scanned 16 times.

### 2.8 Evaluation of cytocompatibility of hydrogels

The cytocompatibility and hemocompatibility of the hydrogels were evaluated using CCK-8 assay, live/dead staining, and hemolysis assay. Hydrogel extracts were first prepared before the CCK-8 assay, live/dead staining, and hemolysis assay. Briefly, freeze-dried hydrogel samples were immersed in complete culture medium or sterile normal saline at a mass-to-volume ratio of 0.2 g/mL and incubated at 37 °C for 24 h. The extracts prepared in complete culture medium were used for the CCK-8 assay and live/dead staining, whereas those prepared in sterile normal saline were used for the hemolysis assay. For hemolysis testing, the original saline extracts were further diluted to 2% (v/v) with normal saline before use.

For the CCK-8 assay, L929 cells were seeded at a density of 5 × 10^4^ cells/sample and co-cultured with the hydrogel extracts at 37 °C for 24 h. Subsequently, CCK-8 solution was added, and the absorbance at 450 nm was recorded using a microplate reader to evaluate cell viability.

For live/dead staining, L929 cells were co-cultured with the hydrogel extracts at 37 °C for 24 h and then stained using a live/dead cell staining kit according to the manufacturer’s instructions. Cell morphology and viability were observed by confocal laser scanning microscopy (CLSM; LSM980, Zeiss, Oberkochen, Germany).

For the hemolysis assay, fresh erythrocytes were washed with normal saline and diluted to obtain a 4% (v/v) erythrocyte suspension. Subsequently, the erythrocyte suspension was incubated with the 2% hydrogel extract at 37 °C for 3 h. Erythrocytes treated with normal saline and 1% Triton X-100 were used as the negative and positive controls, respectively. After incubation, the samples were centrifuged at 2000 rpm for 15 min. The supernatant was photographed, and its absorbance was measured at 545 nm. The hemolysis ratio was calculated according to the following equation:

$$\text{Hemolysis ratio }\left(\%\right)=\frac{OD_{t}-OD_{n}}{OD_{p}-OD_{n}}\times 100\%$$

where $OD_{t}$ represents the hydrogel-treated group, $OD_{n}$ represents the negative control group treated with erythrocyte suspension diluted in normal saline, and $OD_{p}$ represents the positive control group treated with erythrocyte suspension containing 1% Triton X-100.

### 2.9 Reactive oxygen species scavenging ability of hydrogels

The antioxidant activity of the hydrogels was evaluated using a DPPH radical scavenging assay. DPPH assay: Two milliliters of sample solution (2 mg/mL) was mixed with 2 mL of DPPH solution (0.1 mg/mL, prepared in 95% ethanol) and allowed to react for 30 min at room temperature in the dark. The absorbance of the reaction mixture was then measured at 517 nm. The DPPH scavenging rate was calculated as follows:

$$\text{DPPH scavenging rate }\left(\%\right)=\frac{A_{0}-A}{A_{0}}\times 100\%$$

where A represents the absorbance of the sample, and $A_{0}$ represents the absorbance of a mixture of 2 mL of 95% ethanol and 2 mL of 0.1 mg/mL DPPH solution.

### 2.10 *In Vitro* antibacterial activity of hydrogels

*Staphylococcus aureus* (*Staphylococcus aureus*) and *E. coli* (*Escherichia coli*) were selected as representative bacteria to evaluate the antibacterial activity of the hydrogels. Briefly, 10 μL of bacterial suspension (10^7^ CFU/mL) was added to 0.2 g of hydrogel and incubated at 37 °C for 2 h. As a control, 10 μL of bacterial suspension was added to 0.2 g of PBS. The bacterial suspension was then collected, serially diluted, and cultured on agar plates at 37 °C for 24 h. Bacterial survival was calculated according to the following equation:

$$\text{Survival rate }\left(\%\right)=\frac{\text{colony}\;\text{count}\;\text{in}\;\text{hydrogel}\;\text{group}}{\text{colony}\;\text{count}\;\text{in}\;\text{control}\;\text{group}}\times 100\%$$

### 2.11 *In Vitro* anti-inflammatory evaluation

RAW264.7 macrophages (1 × 10^5^cells/well) were seeded into each group, including the blank, H(CODEX), and H(CODEXC) groups, and incubated overnight at 37 °C to allow cell adhesion. Subsequently, RAW264.7 macrophages in each group were stimulated with lipopolysaccharide (LPS, 100 ng/mL) and interferon (20 ng/mL) for 24 h to induce M1 polarization. Cells cultured on standard culture dishes served as the control group. The cells were fixed with 4% paraformaldehyde, permeabilized with 0.5% Triton X-100, and blocked with 1% bovine serum albumin. After staining with iNOS/DAPI and Arg1/DAPI, the cells were observed under a confocal laser scanning microscope.

### 2.12 Cell scratch assay

The effect of hydrogel extracts on fibroblast migration was evaluated using a cell scratch assay. Briefly, L929 cells were seeded in 6-well plates and cultured until reaching approximately 90% confluence. A straight scratch was created in the cell monolayer using a sterile 200-μL pipette tip. After washing with PBS to remove detached cells, the cells were divided into three groups: the Control group, H(CODEX) group, and H(CODEXC) group. Cells in the Control group were treated with complete culture medium, whereas cells in the H(CODEX) and H(CODEXC) groups were treated with the corresponding hydrogel extracts. Images of the scratch area were captured at 0 and 24 h using an inverted microscope. The migrated area rate was quantified using ImageJ and calculated according to the following equation:

$$\text{Migrated area rate }\left(\%\right)=\left[\left(A_{0}-A_{24}\right)\;/\;A_{0}\right]\times 100\%$$

Where A_0_ represents the initial scratch area at 0 h and A_24_ represents the remaining scratch area at 24 h.

### 2.13 Regulation of key cellular behaviors

After 3 days of culture, total RNA was extracted from L929 and RAW264.7 cells grown on the hydrogels using an RNA purification kit and reverse-transcribed using a reverse transcription kit (ABM, Vancouver, Canada). Quantitative real-time PCR was then performed using SYBR Green Realtime PCR Master Mix on a CFX96TM real-time PCR system. The expression levels of the target genes were calculated using the 2^−ΔΔCT^ method and normalized to the housekeeping gene GAPDH. The primer sequences for Col I, Col III, TNF-α, iNOS, IL-10, and Arg1 are listed in Table 1.

**TABLE 1:** Primer sequences.

Gene	Primer sequences (5′-3′)	
GAPDH	Forward	GAA​GGG​CAT​CTT​GGG​CTA​CAC
	Reverse	GTT​GTC​ATT​GAG​AGC​AAT​GCC​A
Col I	Forward	TAG​AGG​CTC​TGA​AGG​TCC​CC
	Reverse	CAC​CAG​CAA​TAC​CAG​GAG​CA
Col III	Forward	CAG​CCA​GGT​CGA​GAT​GGA​TC
	Reverse	CAG​GGC​CAG​TTT​CTC​CTC​TG
TNF-α	Forward	GGC​AGG​TTC​TGT​CCC​TTT​CA
	Reverse	TCT​TCT​GCC​AGT​TCC​ACG​TC
iNOS	Forward	GCC​AAC​ATG​CTA​CTG​GAG​GT
	Reverse	TCC​AGG​ATG​TTG​TAG​CGC​TG
IL-10	Forward	CCA​AGC​CTT​ATC​GGA​AAT​GA
	Reverse	TCC​TGA​GGG​TCT​TCA​GCT​TC
Arg1	Forward	TTT​CTC​AAA​AGG​ACA​GCC​TCG
​	Reverse	ACA​GAC​CGT​GGG​TTC​TTC​AC

### 2.14 *Staphylococcus aureus*-infected wound model in mice

To investigate the wound-healing efficacy of the hydrogels, an *S. aureus*-infected wound model was established in 6-week-old BALB/c mice (body weight, approximately 20 g) supplied by Beijing Vital River Laboratory Animal Technology Co., Ltd (Beijing, China). A total of nine mice were randomly divided into three groups (n = 3 per group). After routine disinfection, full-thickness excisional wounds with a diameter of 10 mm were created on the dorsal region using a sterile biopsy punch. All experimental procedures and outcome assessments were conducted in a blinded manner. The mice were anesthetized by isoflurane inhalation. Briefly, the mice were placed in an induction chamber and anesthetized with 3%–4% isoflurane for induction, followed by maintenance with 1.5%–2.0% isoflurane via a face mask. Under anesthesia, the dorsal hair was shaved and the skin was disinfected, after which a full-thickness circular wound with a diameter of 10 mm was created on the back using a sterile biopsy punch. The wounds were infected with *S. aureus* (10 μL, 1 × 10^7^ CFU/mL) for 24 h before treatment. Infected wounds without any treatment served as the blank control group, whereas the other two groups were treated with H(CODEX) and H(CODEXC), respectively. The hydrogel dressings were replaced every 2 days to ensure sustained drug delivery and promote wound healing during the treatment period. To evaluate wound closure, photographs of the wounds were taken and body weights were recorded on days 0, 3, 6, 9, and 12. Wound areas were quantified using ImageJ and expressed as the percentage of the initial wound area of each individual mouse at day 0, thereby reducing the influence of baseline differences among animals. All animal experiments were conducted in strict accordance with the regulations of the Animal Ethics Committee of Peking University People’s Hospital (2024PHE022).

### 2.15 Histological analysis

For histological analysis, the entire wound tissue together with the surrounding skin was excised on day 12, fixed in 2.5% formaldehyde solution, embedded in paraffin, and sectioned. The samples were then subjected to hematoxylin and eosin (H&E) staining and Masson’s trichrome staining.

### 2.16 Statistical analysis

Data are presented as the mean ± standard deviation (SD). Statistical analysis was performed using GraphPad Prism 9.0. Differences among groups were analyzed by one-way analysis of variance (ANOVA) followed by Tukey’s *post hoc* test. A value of p < 0.05 was considered statistically significant, and p < 0.01 was considered highly significant.

## 3 Results

### 3.1 Preparation and characterization of H(CODEX) and H(CODEXC) hydrogels

In this study, double-network hydrogels were prepared from methacrylated carboxymethyl chitosan (CMCSMA) and oxidized dextran (ODex) through free-radical polymerization and Schiff base formation. Caffeic acid (CA) was then incorporated to generate the H(CODEX) and H(CODEXC) hydrogel systems. This design combines a stable covalently crosslinked network with a dynamic, reversible Schiff base network, thereby providing structural robustness while enabling the loading and controlled release of bioactive molecules.

**FIGURE 2:**
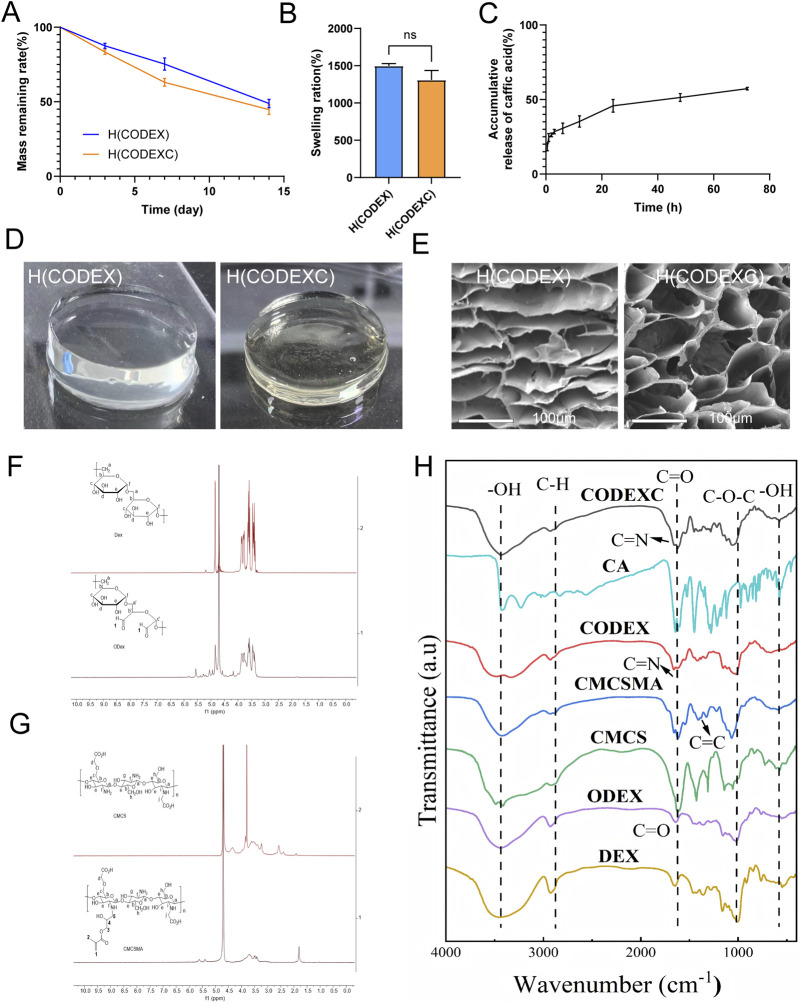
Construction and characterization of H(CODEX) and H(CODEXC) hydrogels **(A)** Degradation behavior of the double-network hydrogels; **(B)** swelling behavior of the double-network hydrogels; **(C)** CA release profile of H(CODEXC); **(D)** macroscopic images of the double-network hydrogels; **(E)** SEM images of the double-network hydrogels; **(F)** ^1^H NMR spectra of DEX and ODex **(G)** ^1^H NMR spectra of CMCS and CMCSMA; **(H)** FTIR spectra of DEX, ODex, CMCS, CMCSMA, CODEX, CA, and CODEXC.

According to the 15-day *in vitro* degradation study, H(CODEXC) degraded more rapidly and to a greater extent than H(CODEX), indicating higher degradability. This feature promotes the release of active molecules and enables the material to better adapt to the dynamic changes in the wound micro environment throughout the healing process. The swelling behavior of the hydrogels was evaluated at 37 °C. H(CODEX) and H(CODEXC) exhibited swelling ratios of 1,498% ± 32.19% and 1,309% ± 126.1%. Both hydrogels showed high swelling and fluid-absorption capacities, suggesting strong potential for wound exudate uptake, maintenance of a moist healing environment, and mitigation of the adverse effects of excessive exudate accumulation on tissue repair. Compared with H(CODEX), H(CODEXC) showed a slightly lower swelling ratio, indicating that CA loading influenced the fluid-absorption behavior of the hydrogel. Together with the subsequent scanning electron microscopy (SEM) observations, this difference is related to a smaller pore size and a denser internal network. Nevertheless, H(CODEXC) retained a relatively high degree of swelling, indicating that, despite its drug-loading capability, it can still meet the basic fluid-management requirements of wound dressings (Figures 2A,B).

To evaluate the drug-delivery performance of H(CODEXC), the *in vitro* release behavior of CA was examined. CA was released from H(CODEXC) in a relatively slow and sustained manner. An initial burst release was observed during the first hour, with a cumulative release of approximately 30%, followed by a markedly reduced release rate, and the cumulative release remained below 60% after 72 h. This release profile, characterized by an early burst followed by a slower release phase, suggests that H(CODEXC) may provide rapid initial CA availability and relatively prolonged release under *in vitro* conditions. This pattern may be favorable for maintaining local bioactivity and supporting its potential antibacterial, antioxidant, and inflammation-modulating effects during infected wound repair (Figure 2C).

In this study, a drug-free double-network hydrogel, H(CODEX), and a CA-loaded double-network hydrogel, H(CODEXC), were successfully prepared. Both hydrogels were transparent, whereas H(CODEXC) exhibited a pale yellow color because of CA incorporation. SEM further showed that both hydrogels possessed a typical honeycomb-like porous microstructure with well-defined pore boundaries, suggesting relatively uniform internal network morphology. Such porous architectures are generally favorable for water uptake, nutrient exchange, and local drug diffusion and are therefore considered desirable morphological features of wound-repair materials. Compared with H(CODEX), H(CODEXC) exhibited smaller pores, indicating a more compact internal network. This observation is consistent with the lower swelling ratio of H(CODEXC) and provides morphological support for the notion that CA loading induced network rearrangement. A denser microstructure can enhance structural stability while partially retarding the diffusion of active molecules, thereby providing a structural basis for the subsequent sustained-release behavior (Figures 2D,E).

Through ^1^H NMR and FTIR, the successful modification of ODex and CMCSMA and the formation of H(CODEXC) were verified. In the ^1^H NMR spectrum of ODex, the characteristic aldehyde proton signal was observed at 9.20 ppm, and the characteristic hemiacetal proton H-c’ signal of the aldehyde-containing sugar ring was observed at 5.65 ppm, proving that DEX was successfully oxidized to generate ODex (Figure 2F). In the ^1^H NMR spectrum of CMCSMA, characteristic vinyl proton signals of the methacryloyl group appeared at 5.71 and 5.49 ppm. In addition, the chemical shift of the allylic methyl proton H-2 appeared at 1.90 ppm (s, H-2); the chemical shifts of the nitrogen-linked methylene proton H-5, oxygen-linked methine proton H-4, and oxygen-linked methylene proton H-3 appeared at 3.58 ppm (s, H-5), 3.52 ppm (s, H-4), and 3.50 ppm (s, H-3), respectively. These characteristic proton signals indicated that the MA segment was successfully grafted onto the CMCS molecular chain (Figure 2G).

FTIR results further showed that an aldehyde-related C=O absorption peak appeared in ODex, and a methacryloyl-related C=C absorption peak appeared in CMCSMA, further confirming the successful preparation of the two precursors. After hydrogel formation, a characteristic C=N absorption peak appeared in the CODEX spectrum, suggesting that the amino groups of CMCSMA reacted with the aldehyde groups of ODex through a Schiff base reaction to form dynamic imine bonds, thereby proving the successful construction of the Schiff base-containing double-network hydrogel. After CA loading, H(CODEXC) retained the main characteristic peaks of CODEX and showed CA-related aromatic ring and phenolic hydroxyl absorption signals, indicating that CA was successfully loaded into the hydrogel network (Figure 2H).

Overall, ^1^H NMR and FTIR analyses confirmed the successful preparation of ODex and CMCSMA, Schiff base-containing double-network formation, and CA loading in H(CODEXC). Together with its favorable swelling capacity, relatively faster degradation, denser porous structure, and sustained CA release, these results demonstrate that H(CODEXC) possesses suitable physicochemical and structural properties for exudate management, local drug delivery, and infected wound microenvironment regulation.

### 3.2 *In Vitro* biocompatibility of H(CODEXC) hydrogel

**FIGURE 3:**
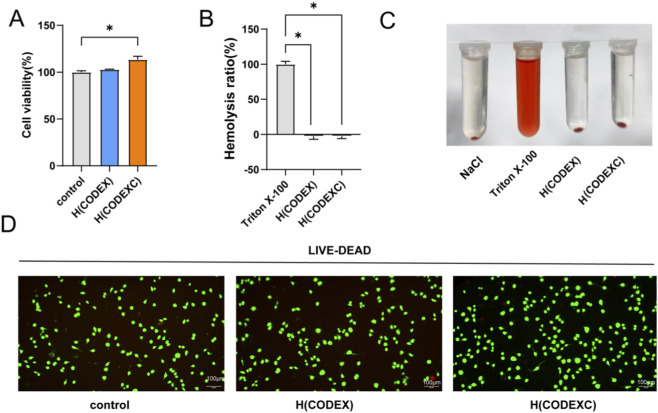
Evaluation of the *in vitro* biocompatibility and hemocompatibility of the hydrogels **(A)** Effects of different treatment groups on the viability of L929 mouse fibroblasts determined by CCK-8 assay; **(B)** hemolysis rates of different treatment groups; **(C)** representative photographs of the supernatants after centrifugation in the hemolysis assay; **(D)** live/dead staining results of L929 mouse fibroblasts in different treatment groups, where green fluorescence indicates live cells and red fluorescence indicates dead cells (n = 6, *, *P < 0.05*).

Good biocompatibility is a prerequisite for the safe use and reparative function of wound dressings. To evaluate the cytocompatibility of the constructed hydrogels, their interactions with L929 mouse fibroblasts were assessed using the CCK-8 assay and live/dead staining. After 24 h of culture, cell viability in the H(CODEXC) group was significantly higher than that in the blank control group, indicating good cytocompatibility and a capacity to promote fibroblast proliferation to some extent. The live/dead staining results further supported this finding: green fluorescent live cells predominated in all hydrogel-treated groups, and most cells displayed a typical spindle-shaped, well-spread morphology, whereas relatively few red fluorescent dead cells were observed. These results indicate that none of the materials exhibited obvious cytotoxicity. Because fibroblasts are essential for collagen secretion, extracellular matrix deposition, and granulation tissue formation, these findings suggest that the hydrogel system provides a favorable microenvironment for wound-repair-related cells and thereby supports subsequent tissue reconstruction (Figures 3A,D).

Hemocompatibility was further evaluated using a hemolysis assay. The hemolysis rates of both H(CODEX) and H(CODEXC) were below 5% and were significantly lower than that of the Triton X-100 positive control. Representative post-centrifugation photographs showed that, except for the Triton X-100 group, the supernatants in all other groups remained clear and transparent, with no obvious hemoglobin release. These results indicate that this series of hydrogels exerts minimal effects on erythrocyte membrane integrity and exhibits good hemocompatibility. Because wound dressings inevitably come into contact with bleeding wound surfaces and tissue fluid in practical applications, these findings support the safety of the constructed hydrogels for local use and provide a biological basis for their further development as dressings for infected wounds (Figures 3B,C).

### 3.3 *In Vitro* antibacterial activity of H(CODEXC) hydrogel

To evaluate the antibacterial activity of H(CODEXC), Gram-negative *Escherichia coli* and Gram-positive *Staphylococcus aureus* were used as model bacterial strains. Compared with the control group, bacterial survival rates were lower in all hydrogel-treated groups, indicating that the prepared hydrogels possessed antibacterial activity. Among them, H(CODEXC) showed the strongest inhibitory effect against both strains, suggesting that CA loading further enhanced the *in vitro* antibacterial performance of the hydrogel. These results indicate that H(CODEXC) can reduce bacterial burden and create a more favorable local environment for the treatment of infected wounds.

**FIGURE 4:**
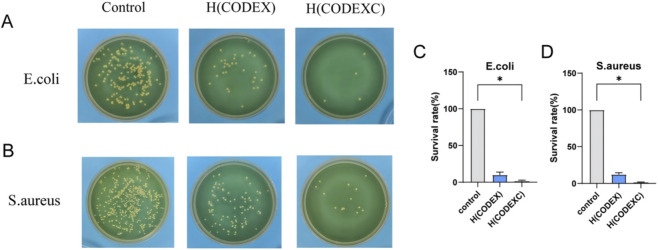
Evaluation of the *in vitro* antibacterial activity of the hydrogels. **(A,B)** Colony-forming plate images of *E. coli* and *S. aureus* after treatment with different groups; **(C,D)** Quantitative analysis of the survival rates of *E. coli* and *S. aureus* after different treatments. (n = 6, *P < 0.05).

The double-network hydrogel exhibited intrinsic antibacterial activity, and CA loading further improved inhibition of both Gram-positive and Gram-negative bacteria, suggesting its potential for broad-spectrum antibacterial applications. In infected wounds, a high local bacterial burden promotes persistent inflammation, aggravates tissue injury, and delays healing. Therefore, the enhanced antibacterial performance of H(CODEXC) helps limit bacterial colonization within the wound microenvironment and create more favorable conditions for inflammation resolution and tissue repair. Overall, these *in vitro* results support the potential application of H(CODEXC) as a dressing for infected wounds (Figure 4).

### 3.4 Repair-promoting activity of H(CODEXC) hydrogel

**FIGURE 5:**
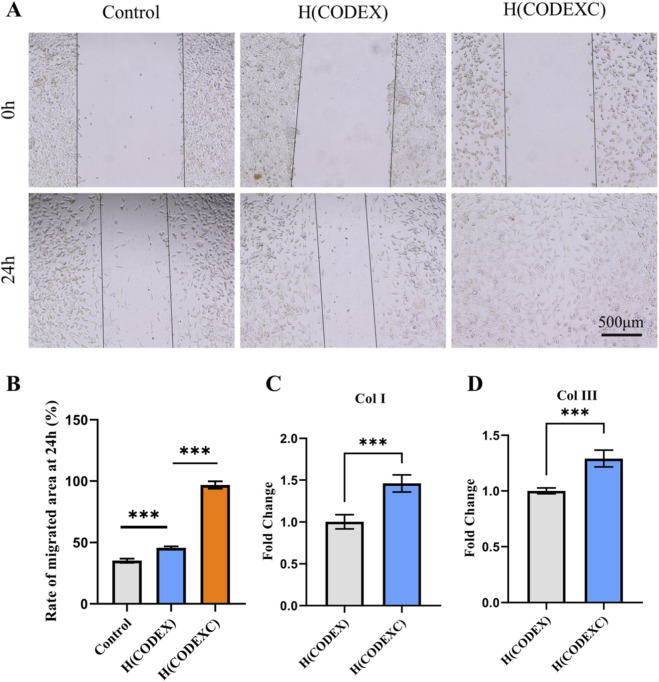
Effects of H(CODEXC) on L929 cell migration and collagen-related gene expression **(A)** Representative images of L929 cell scratch closure in the Control, H(CODEX), and H(CODEXC) groups at 0 and 24 h. Scale bar: 500 μm **(B)** Quantitative analysis of the migrated area rate at 24 h; **(C,D)** Relative expression levels of Col I and Col III in L929 cells. Data are presented as mean ± SD (n = 6, *P < 0.05, **P < 0.01, ***P < 0.001).

To evaluate the effect of H(CODEXC) on fibroblast-associated repair behaviors, L929 cell migration was first assessed using a scratch assay. After 24 h of treatment, the scratch area was partially closed in all groups; however, the H(CODEXC) group showed the most pronounced wound closure compared with the Control and H(CODEX) groups. Quantitative analysis further confirmed that the migrated area rate at 24 h was significantly higher in the H(CODEXC) group, indicating that CA loading enhanced the ability of the hydrogel extract to promote L929 cell migration (Figures 5A,B).

To further investigate whether H(CODEXC) affected extracellular matrix-related gene expression, qRT-PCR was performed to detect the expression of Col I and Col III in L929 cells. Compared with the H(CODEX) group, H(CODEXC) significantly upregulated both Col I and Col III expression. Since type I and type III collagen are important components of the extracellular matrix during wound repair, these results suggest that H(CODEXC) may support fibroblast-mediated matrix remodeling by promoting both cell migration and collagen-related gene expression (Figures 5C,D).

### 3.5 Antioxidant activity of H(CODEXC) hydrogel

**FIGURE 6:**
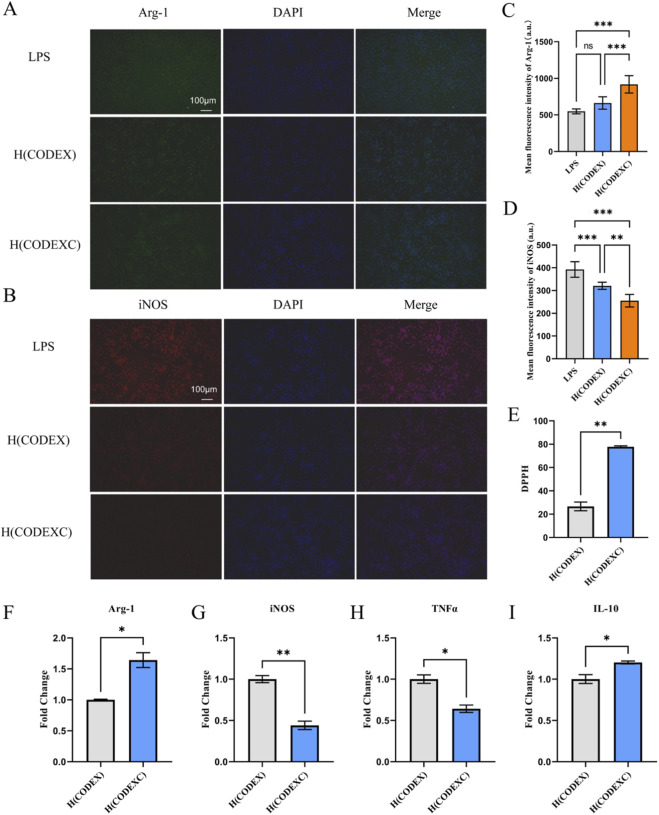
Immunomodulatory and antioxidant effects of the hydrogels. **(A)** Representative immunofluorescence images of Arg-1 in RAW264.7 macrophages **(B)** Representative immunofluorescence images of iNOS in RAW264.7 macrophages **(C,D)** Quantitative analysis of the mean fluorescence intensity of Arg-1 and iNOS **(E)** DPPH free-radical-scavenging capacity of H(CODEX) and H(CODEXC) **(F–I)** Relative expression levels of Arg-1, iNOS, TNF-α, and IL-10 in RAW264.7 macrophages. Data are presented as mean ± SD (n = 6, *P < 0.05, **P < 0.01, ***P < 0.001).

Inflammatory dysregulation and oxidative stress are key pathological factors in delayed healing of infected wounds. Macrophage polarization directly shapes the local inflammatory microenvironment, whereas excessive accumulation of reactive oxygen species (ROS) further exacerbates tissue damage and impairs repair. On this basis, the biological effects of the CA-loaded hydrogel were evaluated in terms of macrophage phenotype modulation and free-radical-scavenging capacity. Immunofluorescence showed that, compared with the control (LPS) group, Arg-1 expression in RAW264.7 macrophages was significantly upregulated in both the H(CODEX) and H(CODEXC) groups. Meanwhile, iNOS expression was significantly downregulated in the H(CODEXC) group relative to the control group. Consistent with these quantitative results, the LPS group exhibited weak green fluorescence for Arg-1 and strong red fluorescence for iNOS, whereas hydrogel treatment, particularly H(CODEXC), produced a more pronounced increase in Arg-1 signal and decrease in iNOS signal. These findings suggest that the CA-loaded hydrogel more effectively promotes the shift of macrophages from a pro-inflammatory phenotype to an anti-inflammatory/pro-repair phenotype, thereby improving the inflammatory status of infected wounds (Figures 6A–D).

The *in vitro* antioxidant capacity of the hydrogels was further evaluated using a DPPH free-radical-scavenging assay. H(CODEX) exhibited a DPPH scavenging rate of approximately 30%, whereas H(CODEXC) reached approximately 80%, which was significantly higher than that of the CA-free hydrogel. These data indicate that CA incorporation markedly enhanced the free-radical-scavenging capacity of the material and conferred superior antioxidant potential. Together, these results demonstrate that H(CODEXC) can not only modulate macrophage phenotype and attenuate pro-inflammatory responses but also effectively scavenge free radicals and reduce oxidative-stress-related damage, thereby creating a more favorable environment for cell survival, microenvironmental homeostasis, and wound repair (Figure 6E).

To further investigate the effects of the hydrogels on inflammation-related molecular expression, qRT-PCR was performed in RAW264.7 macrophages after different treatments. Compared with the H(CODEX) group, the H(CODEXC) group exhibited significantly increased expression of Arg-1 and IL-10, both of which are commonly associated with anti-inflammatory and reparative macrophage responses. In contrast, the expression levels of iNOS and TNF-α were significantly decreased after H(CODEXC) treatment. These results suggest that CA loading further enhanced the inflammation-modulating capacity of the hydrogel, shifting macrophage-related molecular responses toward a more anti-inflammatory and repair-favorable profile (Figures 6F–I).

This trend was consistent with the immunofluorescence results, in which H(CODEXC) increased Arg-1 fluorescence intensity and reduced iNOS fluorescence intensity compared with H(CODEX). The consistency between protein-level immunofluorescence analysis and gene-level qRT-PCR results strengthens the evidence that H(CODEXC) can regulate macrophage phenotype-related responses. Given the important role of macrophage-mediated inflammation in infected wound repair, the upregulation of Arg-1 and IL-10 together with the downregulation of iNOS and TNF-α indicates that H(CODEXC) may help attenuate excessive inflammatory activation and create a more favorable microenvironment for subsequent tissue repair. These findings further support the contribution of CA incorporation to the immunomodulatory function of the double-network hydrogel.

### 3.6 Evaluation of the in vivo wound-healing effect of H(CODEXC) hydrogel

**FIGURE 7:**
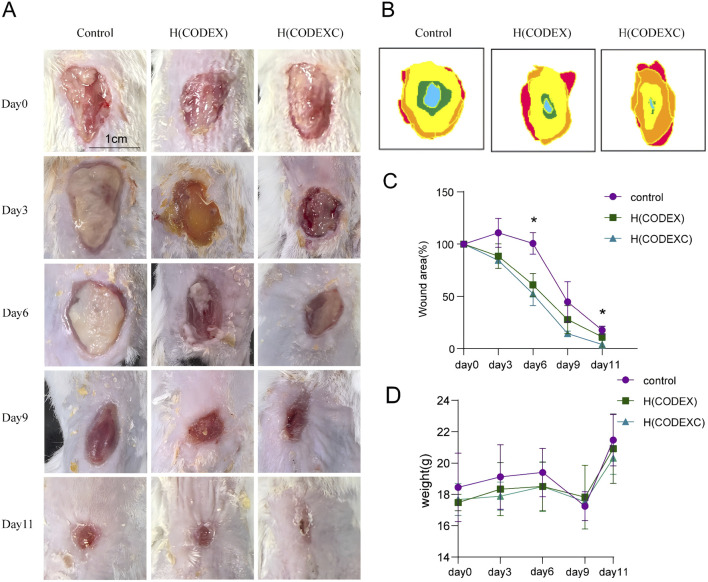
Effects of different treatment groups on the healing of infected full-thickness skin wounds **(A)** Representative gross images of wounds in the control, H(CODEX), and H(CODEXC) groups at different time points; **(B)** overlay schematic of wound-area changes in each group; **(C)** quantitative analysis of the percentage of wound area over time; **(D)** body-weight changes (n = 3, *P < 0.05).

To verify the pro-reparative effect of the material, a mouse model of infected full-thickness skin defects was established, and wound healing was monitored dynamically across treatment groups. Gross examination showed that wound areas gradually decreased over time in all groups; however, marked differences in wound appearance and healing progression were evident. In the control group, obvious purulent exudate and wound crusting remained visible from day 0 to day 6, suggesting poor local infection control and relatively slow contraction of the wound margins. By days 9 and 12, although the wound area had decreased relative to earlier time points, sizeable unhealed regions persisted. In contrast, neither the H(CODEX) nor H(CODEXC) group showed obvious purulent exudate during the early stage, and local wound conditions were markedly improved relative to the control. Among the treated groups, H(CODEXC) showed the most pronounced effect, with a cleaner wound bed and more evident contraction of the wound margins. From day 6 onward, wound area decreased substantially, and by days 9 and 12 the residual open area had further diminished, resulting in an overall repair appearance superior to that of both the control and H(CODEX) groups. The wound-area overlay images shown in Figure 7B further illustrate the greater contraction observed over time in the H(CODEXC) group, indicating superior performance in improving local wound status and promoting wound closure (Figures 7A,B).

On this basis, quantitative analysis of wound-area changes was performed. The results showed a gradual reduction in wound area over time in all groups, with different degrees of wound closure among the treatment groups. Significant intergroup differences were observed on days 6 and 12, suggesting that the treatments had different effects on the healing progression of infected wounds. Specifically, on day 6, wound areas in both the H(CODEX) and H(CODEXC) groups were significantly smaller than that in the control group (P < 0.05), whereas no statistically significant difference was detected between the two hydrogel-treated groups. By day 9, the wound area in the H(CODEXC) group had decreased to 14.4% of the initial area, compared with 27.8% in the control group and 28.0% in the H(CODEX) group; however, these intergroup differences did not reach statistical significance. By day 12, wound areas in the control, H(CODEX), and H(CODEXC) groups were 17.7%, 11.4%, and 4.1% of the initial area, respectively. The H(CODEXC) group exhibited a significantly smaller residual wound area than the control group (P < 0.05), although the difference relative to the H(CODEX) group remained statistically insignificant. Overall, H(CODEXC) consistently showed smaller residual wound areas at multiple time points, suggesting a superior capacity to promote closure of infected wounds (Figure 7C).

To further evaluate the effects of the different treatments on the quality of tissue repair in infected wounds, wound tissues from each group were analyzed by H&E and Masson staining. H&E staining revealed that, in the control group, obvious tissue defects remained in the central wound area, epidermal continuity was poorly restored, and the wound edges had not fully rejoined. In addition, abundant inflammatory cell infiltration was observed in the dermis, and the local tissue architecture appeared loose, indicating persistent inflammation and incomplete repair. In contrast, the H(CODEX) group showed markedly improved epidermal coverage and more obvious wound contraction than the control group, although some inflammatory cell infiltration and incomplete tissue remodeling were still present locally. The H(CODEXC) group exhibited the most favorable tissue morphology: the wound surface was largely re-epithelialized, the epidermal layer was continuous and relatively intact, the tissue was more densely and orderly arranged, and inflammatory cell infiltration was markedly reduced relative to both the control and H(CODEX) groups. These findings suggest that H(CODEXC) more effectively promotes the transition of infected wounds from the inflammatory phase to the tissue-reconstruction phase.

**FIGURE 8:**
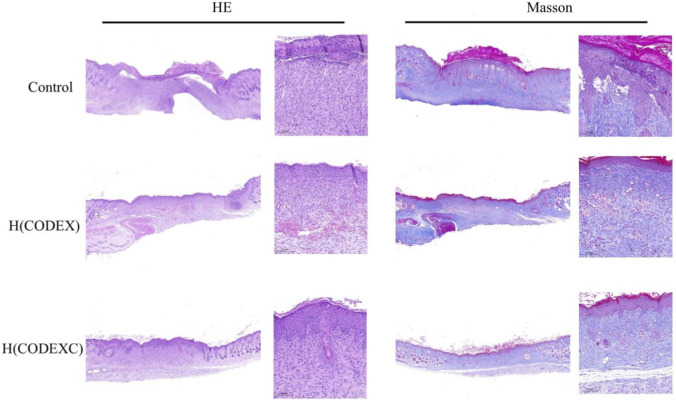
H&E and Masson staining results of infected full-thickness skin wounds in different treatment groups.

Masson staining further revealed clear intergroup differences in collagen deposition and matrix remodeling. In the control group, collagen staining was relatively weak, with sparse and disorganized fibers, indicating insufficient extracellular matrix reconstruction. In the H(CODEX) group, the area of blue-stained collagen was greater than that in the control group, suggesting enhanced collagen deposition and granulation tissue formation, although the overall fiber arrangement remained relatively loose. By contrast, the H(CODEXC) group showed more abundant collagen deposition, a larger blue-stained area, and more continuous, compact, and orderly fiber bundles, indicating that the CA-loaded hydrogel was more conducive to extracellular matrix reconstruction and mature wound repair. Collectively, the H&E and Masson results showed that H(CODEXC) outperformed both the control and H(CODEX) groups in promoting re-epithelialization, reducing inflammatory cell infiltration, and enhancing collagen deposition and tissue remodeling. These histological findings are consistent with the gross wound observations and quantitative wound-area analysis, further demonstrating that the CA-loaded double-network hydrogel can more effectively improve the local microenvironment of infected wounds and promote tissue repair (Figure 8).

## 4 Discussion

The repair of infected wounds is often hindered by multiple adverse factors, including persistent bacterial colonization, dysregulated inflammatory responses, and excessive oxidative stress. Therefore, interventions that rely solely on physical coverage or the action of a single drug are insufficient to achieve effective treatment of infected wounds (Hunt et al., 2024; Hossei et al., 2025). Based on these considerations, a double-network hydrogel was selected as the material platform in this study. Effective treatment of infected wounds requires not only coverage of the wound surface, exudate absorption, and maintenance of a moist local environment, but also adequate structural stability under complex local conditions and the ability to deliver bioactive molecules. Compared with single-network systems, double-network hydrogels are generally better able to balance mechanical performance, network stability, environmental adaptability, and drug-loading capacity, making them more suitable for infected wound treatment involving exudate management, microenvironment regulation, and sustained drug release (Liu et al., 2025). On this basis, caffeic acid was further selected as the functional bioactive molecule because its biological activities closely match the core therapeutic demands of infected wound repair. Caffeic acid possesses antioxidant, anti-inflammatory, and antibacterial properties, allowing it to simultaneously target several key pathological events, including elevated bacterial burden, excessive oxidative stress, and disruption of the inflammatory microenvironment (Zhang et al., 2024). Accordingly, loading caffeic acid into a double-network hydrogel not only helps exploit the structural support and local delivery advantages of the material itself, but also enables a more effective integration of antibacterial, antioxidant, and inflammation-regulating functions, thereby providing a more rational strategy for the comprehensive treatment of infected wounds. In addition, as a structurally defined small-molecule phenolic acid, caffeic acid is more amenable to controlled loading, release, and mechanistic investigation. Since studies using caffeic acid as an independent active molecule in hydrogel systems for infected wound treatment remain relatively limited, this work also fills, to some extent, a gap in this research area.

Therefore, a caffeic acid-loaded double-network hydrogel, H(CODEXC), was constructed in this study and was found to promote wound healing more effectively than the blank double-network hydrogel, H(CODEX). This system does not rely solely on the passive protective function of the material itself. Instead, it integrates the structural support of the double-network hydrogel with the biological activities of caffeic acid to synergistically improve the local microenvironment of infected wounds through antibacterial, antioxidant, and inflammation-regulating effects, thereby promoting wound repair. The results showed that, while maintaining a favorable porous structure, fluid absorption capacity, and biocompatibility, H(CODEXC) further enhanced the inhibition of *Staphylococcus aureus* and *Escherichia coli*, improved free-radical-scavenging ability, and exhibited superior *in vivo* effects in terms of infection control, wound closure, re-epithelialization, and collagen deposition. These findings indicate that the incorporation of caffeic acid confers enhanced biological functionality on the double-network hydrogel and highlight the importance of synergistic interactions between material structure and bioactive molecules in wound repair.

From the perspective of material behavior, the pro-reparative potential of H(CODEXC) may be associated, at least in part, with its CA release profile. The *in vitro* release results obtained under PBS conditions showed that H(CODEXC) exhibited an initial burst release followed by a slower release phase. This release pattern may be favorable for wound repair, as the early release phase could provide rapid CA availability at the initial stage of treatment, while the subsequent slower release phase may help maintain local CA exposure over a relatively prolonged period under *in vitro* conditions. Such a profile may support the antibacterial, antioxidant, and inflammation-modulating potential of H(CODEXC), thereby contributing to the improvement of the infected wound microenvironment.

Delayed healing of infected wounds is driven not only by an increased bacterial burden, but also by persistent dysregulation of the inflammatory microenvironment (Mu et al., 2025; Li W. et al., 2025). Therefore, this study further examined the regulatory effects of H(CODEXC) on inflammation-related cellular behaviors. Immunofluorescence results showed that H(CODEXC) increased Arg-1 expression and reduced iNOS expression in macrophages, suggesting that the CA-loaded hydrogel modulated macrophage phenotype-related responses toward a more anti-inflammatory and repair-associated profile. This tendency was further supported by qRT-PCR analysis, which showed that H(CODEXC) upregulated the expression of Arg-1 and IL-10 while downregulating iNOS and TNF-α. The consistency between protein-level immunofluorescence observations and gene-level qRT-PCR results strengthens the evidence that CA incorporation contributes to the immunomodulatory function of the hydrogel. In infected wounds, excessive and prolonged inflammatory activation can impair tissue repair by aggravating local cell damage, delaying fibroblast function, and interfering with extracellular matrix remodeling. Therefore, the ability of H(CODEXC) to suppress pro-inflammatory markers while enhancing anti-inflammatory and repair-associated markers may help improve the local inflammatory microenvironment and facilitate subsequent tissue regeneration. Together with its antibacterial and antioxidant properties, this inflammation-modulating effect may represent an important mechanism by which H(CODEXC) promotes infected wound healing (Yan et al., 2024; Sun et al., 2024). Meanwhile, the DPPH results demonstrated a markedly enhanced free-radical-scavenging capacity, suggesting that this system can further improve the local microenvironment by reducing oxidative stress. Previous studies have shown that persistent ROS accumulation in infected wounds aggravates tissue damage and amplifies inflammatory responses (Hunt et al., 2024; Sun et al., 2024). In this context, the dual antioxidant and immunoregulatory effects of H(CODEXC) enable the system to synchronously modulate inflammation and oxidative stress while suppressing bacterial growth, thereby creating a more favorable environment for tissue regeneration. In addition to macrophage regulation, the effects of H(CODEXC) on fibroblast-associated repair behaviors further support its multi-level pro-reparative activity. The scratch assay showed that H(CODEXC) promoted L929 cell migration, providing direct *in vitro* functional evidence for its potential role in supporting wound closure. Moreover, qRT-PCR results showed that H(CODEXC) significantly increased the expression of Col I and Col III in fibroblasts, indicating its beneficial effects on extracellular matrix-related gene expression. Since fibroblast migration and collagen-related matrix remodeling are closely involved in granulation tissue formation and wound repair, these findings suggest that H(CODEXC) may contribute to tissue reconstruction by enhancing both cellular migration and matrix-associated reparative responses (Liang et al., 2025; Jin et al., 2025). Enhanced fibroblast activity and collagen deposition were further confirmed by *in vivo* H&E and Masson staining. Compared with the control and H(CODEX) groups, the H(CODEXC) group exhibited more pronounced re-epithelialization, reduced inflammatory cell infiltration, and more abundant, continuously organized collagen deposition. Collectively, these findings indicate that the beneficial effects of H(CODEXC) on infected wounds are not limited to a single aspect, but span bacterial control, oxidative stress relief, inflammatory microenvironment improvement, fibroblast support, and tissue reconstruction, highlighting its strong synergistic reparative potential.

From translational perspective, H(CODEXC) shows promising potential for clinical application. As a bioactive double-network dressing, this system possesses favorable fluid absorption and biocompatibility, allowing it to meet the basic requirements for local wound coverage, exudate management, and maintenance of a moist healing environment. At the same time, caffeic acid loading endows the material with integrated antibacterial, antioxidant, and inflammation-regulating activities. Unlike conventional dressings that mainly serve as passive physical barriers, H(CODEXC) is designed to actively intervene in the dysregulated local microenvironment of infected wounds, which gives it potential advantages in the treatment of complex infected wounds. In addition, by integrating structural support with local drug delivery, this material has potential for further development as a novel functional wound dressing.

Nevertheless, several limitations of this study should be acknowledged. First, the present work was mainly based on *in vitro* experiments and a mouse model of infected wounds, which do not fully recapitulate the pathophysiological complexity of clinical wounds. In addition, the *in vivo* wound-healing experiment was performed with a relatively small animal cohort, with three mice per group. Although the normalized wound-area analysis, gross observations, and histological staining results showed generally consistent pro-reparative trends, future studies with larger sample sizes and independent biological replicates are needed to further confirm the therapeutic efficacy of H(CODEXC). Its applicability to more challenging settings, such as chronic infections, diabetic wounds, and drug-resistant bacterial infections, also requires further validation. Second, the current safety evaluation was largely limited to short-term *in vitro* and *in vivo* biocompatibility, while systematic studies on long-term toxicity, the safety of degradation products, and local tolerance under repeated administration remain lacking. Third, the *in vivo* release behavior, local retention time, and pharmacodynamic characteristics of caffeic acid in the hydrogel have not yet been fully elucidated, and the precise molecular mechanisms underlying its pro-reparative effects require further investigation. In addition, although encouraging findings were obtained regarding inflammation modulation, the current evidence remains mainly limited to phenotypic observations and partial molecular analyses. In particular, the signaling pathways involved in macrophage polarization and inflammation regulation have not yet been systematically validated. Therefore, the mechanistic changes associated with the transition of infected wounds from the inflammatory phase to the regenerative phase still need to be clarified in future studies.

## 5 Conclusion

In this study, a caffeic-acid-loaded double-network hydrogel, H(CODEXC), was successfully constructed. This hydrogel exhibited favorable physicochemical properties, biocompatibility, and sustained drug-release behavior, as well as superior antibacterial and antioxidant activities relative to the drug-free hydrogel. *In vitro* antibacterial experiments showed that H(CODEXC) more effectively inhibited both *S. aureus* and *E. coli*, indicating that CA loading enhanced the broad-spectrum antibacterial performance of the hydrogel. Further analyses demonstrated that H(CODEXC) modulated macrophage phenotype and promoted fibroblast-associated matrix remodeling. *In vivo* experiments further confirmed that this hydrogel improved the local microenvironment of infected wounds and promoted wound closure, re-epithelialization, and collagen deposition. H(CODEXC) represents a promising strategy for the design of bioactive dressings for infected wounds and has considerable potential for local anti-infective therapy, attenuation of oxidative stress, and promotion of tissue repair.

## Data Availability

The original contributions presented in the study are included in the article/supplementary material, further inquiries can be directed to the corresponding authors.

## References

[B1] Andrade M. Benfeito S. Soares P. Magalhaes E Silva D. Loureiro J. (2015). Fine-tuning of the hydrophobicity of caffeic acid: studies on the antimicrobial activity against *Staphylococcus aureus* and *Escherichia coli* . RSC Adv. 5, 53915–53925. 10.1039/c5ra05840f

[B2] Badia J. M. Casey A. L. Petrosillo N. Hudson P. M. Mitchell S. A. Crosby C. (2017). Impact of surgical site infection on healthcare costs and patient outcomes: a systematic review in six European countries. J. Hosp. Infect. 96, 1–15. 10.1016/j.jhin.2017.03.004 28410761

[B3] Benbettaieb N. Nyagaya J. Seuvre A. M. Debeaufort F. (2018). Antioxidant activity and release kinetics of caffeic and p-coumaric acids from hydrocolloid-based active films for healthy packaged food. J. Agric. Food Chem. 66, 6906–6916. 10.1021/acs.jafc.8b01846 29852064

[B4] Dong L. Han Z. Zhang H. Yang R. Fang J. Wang L. (2022). Tea polyphenol/glycerol-treated double-network hydrogel with enhanced mechanical stability and anti-drying, antioxidant and antibacterial properties for accelerating wound healing. Int. J. Biol. Macromol. 208, 530–543. 10.1016/j.ijbiomac.2022.03.128 35346679

[B5] He Y. Li Y. Sun Y. Zhao S. Feng M. Xu G. (2021). A double-network polysaccharide-based composite hydrogel for skin wound healing. Carbohydr. Polym. 261, 117870. 10.1016/j.carbpol.2021.117870 33766357

[B6] He Y. Cen Y. Tian M. (2024). Immunomodulatory hydrogels for skin wound healing: cellular targets and design strategy. J. Mater. Chem. B 12, 2435–2458. 10.1039/d3tb02626d 38284157

[B7] Hosseini S. A. Noruzi S. Kesharwani P. Sahebkar A. (2025). Hydrogel-based dressing for wound healing: a systematic review of clinical trials. Int. J. Biol. Macromol. 308, 142322. 10.1016/j.ijbiomac.2025.142322 40118421

[B8] Hunt M. Torres M. Bachar-Wikstrom E. Wikstrom J. D. (2024). Cellular and molecular roles of reactive oxygen species in wound healing. Commun. Biol. 7, 1534. 10.1038/s42003-024-07219-w 39562800 PMC11577046

[B9] Jin C. Jin Y. Ding Z. Nuch K. S. Han M. Shim J. (2025). Cellular and molecular mechanisms of wound repair: from biology to therapeutic innovation. Cells 14, 1850. 10.3390/cells14231850 41369338 PMC12691224

[B10] Kepa M. Miklasinska-Majdanik M. Wojtyczka R. D. Idzik D. Korzeniowski K. Smolen-Dzirba J. (2018). Antimicrobial potential of caffeic acid against *Staphylococcus aureus* clinical strains. Biomed. Res. Int. 2018, 7413504. 10.1155/2018/7413504 30105241 PMC6076962

[B11] Khan F. Bamunuarachchi N. I. Tabassum N. Kim Y. M. (2021). Caffeic acid and its derivatives: antimicrobial drugs toward microbial pathogens. J. Agric. Food Chem. 69, 2979–3004. 10.1021/acs.jafc.0c07579 33656341

[B12] Li Z. Zhao Y. Huang H. Zhang C. Liu H. Wang Z. (2022). A nanozyme-immobilized hydrogel with endogenous ROS-Scavenging and oxygen generation abilities for significantly promoting oxidative diabetic wound healing. Adv. Healthc. Mater 11, e2201524. 10.1002/adhm.202201524 36100580

[B13] Li M. Tang H. Geng X. Zhou J. Mou S. Li C. (2025a). All-natural hydrogel composed of carboxymethyl chitosan and oxidized dextran for promoting wound healing by immune-microenvironment regulation. Carbohydr. Polym. 347, 122731. 10.1016/j.carbpol.2024.122731 39486961

[B14] Li W. Chen Q. Ma Y. Su H. Ren H. Wang H. (2025b). Antibacterial hydrogels for bacteria-infected wound treatment. Biomed. Technol. 9, 100066. 10.1016/j.bmt.2024.11.001

[B15] Liang R. Pan R. He L. Dai Y. Jiang Y. He S. (2025). Decellularized extracellular matrices for skin wound treatment. Materials 18, 2752. 10.3390/ma18122752 40572885 PMC12194566

[B16] Liu Y. Zhang Y. Jia Q. Liang X. Xu K. (2025). Rapid *in situ* formation of a double cross-linked network hydrogels for wound healing promotion. Front. Pharmacol. 16, 1562264. 10.3389/fphar.2025.1562264 40170721 PMC11959063

[B17] Ma H. Axi Y. Lu Y. Dai C. Huang S. Kong Z. (2024). A dual network cross-linked hydrogel with multifunctional Bletilla striata polysaccharide/gelatin/tea polyphenol for wound healing promotion. Int. J. Biol. Macromol. 265, 130780. 10.1016/j.ijbiomac.2024.130780 38471606

[B18] Meng W. Lin Z. Cheng X. Gou S. Wang R. Bu P. (2024). Thiourea-cation chelation based hydrogel and its application as antibacterial dressing for the repair of diabetic wound. Adv. Funct. Mater. 34, 2314202. 10.1002/adfm.202314202

[B19] Mu W. Wang Y. Liu T. Zhang H. Weng L. Chen X. (2025). Nanocomposite hydrogel to integratively inhibit bacteria and inflammation for infected wound repair. J. Control Release 386, 114145. 10.1016/j.jconrel.2025.114145 40848761

[B20] Nam G. S. Park H. J. Nam K. S. (2020). The antithrombotic effect of caffeic acid is associated with a cAMP-dependent pathway and clot retraction in human platelets. Thromb. Res. 195, 87–94. 10.1016/j.thromres.2020.07.024 32682003

[B21] Ouyang A. Qin X. Su B. Mo S. Jiang J. Wang Z. (2026). Metformin glycyrrhetinic acid binary injectable hydrogel for synergistic tumor immunotherapy via spatiotemporal microenvironment remodeling. Mater. Today Bio 36, 102749. 10.1016/j.mtbio.2025.102749 41560796 PMC12813322

[B22] Pandey K. B. Rizvi S. I. (2009). Plant polyphenols as dietary antioxidants in human health and disease. Oxid. Med. Cell Longev. 2, 270–278. 10.4161/oxim.2.5.9498 20716914 PMC2835915

[B23] Park M. Y. Kang D. H. (2021). Antibacterial activity of caffeic acid combined with UV-A light against *Escherichia coli* O157:H7, *Salmonella enterica* serovar typhimurium, and Listeria monocytogenes. Appl. Environ. Microbiol. 87, e0063121. 10.1128/aem.00631-21 33990307 PMC8276812

[B24] Shang F. Qu Y. Li Y. Dong L. Liu D. Wang Z. (2025). Delivered baicalein immunomodulatory hydrogel with dual properties of pH-responsive and anti-infection orchestrates pro-regenerative response of macrophages for enhanced hypertrophic scar therapy. Mater. Today Bio 34, 102160. 10.1016/j.mtbio.2025.102160 40822925 PMC12355094

[B25] Stanciauskaite M. Poskute M. Kurapkiene V. Marksa M. Jakstas V. Ivanauskas L. (2023). Optimization of delivery and bioavailability of encapsulated caffeic acid. Foods 12, 1993. 10.3390/foods12101993 37238812 PMC10217660

[B26] Stasilowicz-Krzemien A. Rosiak N. Miklaszewski A. Cielecka-Piontek J. (2023). Screening of the anti-neurodegenerative activity of caffeic acid after introduction into inorganic metal delivery systems to increase its solubility as the result of a mechanosynthetic approach. Int. J. Mol. Sci. 24, 9218. 10.3390/ijms24119218 37298169 PMC10252749

[B27] Sun D. Chang Q. Lu F. (2024). Immunomodulation in diabetic wounds healing: the intersection of macrophage reprogramming and immunotherapeutic hydrogels. J. Tissue Eng. 15, 20417314241265202. 10.1177/20417314241265202 39071896 PMC11283672

[B28] Tang Y. Zhao R. Yi M. Ge Z. Wang D. Wang G. (2024a). Multifunctional hydrogel enhances inflammatory control, antimicrobial activity, and oxygenation to promote healing in infectious wounds. Biomacromolecules 25, 2423–2437. 10.1021/acs.biomac.3c01386 38457661

[B29] Tang Z. Dan N. Chen Y. (2024b). Utilizing epoxy Bletilla striata polysaccharide collagen sponge for hemostatic care and wound healing. Int. J. Biol. Macromol. 259, 128389. 10.1016/j.ijbiomac.2023.128389 38000600

[B30] Wang L. Zhou M. Xu T. Zhang X. (2022). Multifunctional hydrogel as wound dressing for intelligent wound monitoring. Chem. Eng. J. 433, 134625. 10.1016/j.cej.2022.134625

[B31] Wang W. Jia B. Xu H. Li Z. Qiao L. Zhao Y. (2023a). Multiple bonds crosslinked antibacterial, conductive and antioxidant hydrogel adhesives with high stretchability and rapid self-healing for MRSA infected motion skin wound healing. Chem. Eng. J. 468, 143362. 10.1016/j.cej.2023.143362

[B32] Wang G. Yang F. Zhou W. Xiao N. Luo M. Tang Z. (2023b). The initiation of oxidative stress and therapeutic strategies in wound healing. Biomed. Pharmacother. 157, 114004. 10.1016/j.biopha.2022.114004 36375308

[B33] Wang C. Lu Q. Xiang Y. Yin Y. Li J. Liu Y. (2023c). Enhanced biocompatibility of silk sericin/caffeic acid nanoparticles by red blood cell membranes cloaking. Int. J. Biol. Macromol. 238, 124133. 10.1016/j.ijbiomac.2023.124133 36963548

[B34] Wang J. Wang J. Zhou H. Ma R. Fang Z. Zhu J. (2024). Universal antibacterial and anti-inflammatory treatment using chitosan-prussian blue nanozyme. EngMedicine 1, 100006. 10.1016/j.engmed.2024.100006

[B35] Xiong Q. Q. Chen M. J. Cheng S. Yu J. Ahmad M. Luo H. (2025). Hypericum aqueous extract promotes skin wound healing by modulating the PI3K/Akt signaling pathway. Food and Med. Homol. 3, 9420125. 10.26599/FMH.2026.9420125

[B36] Yan L. Wang J. Cai X. Liou Y. C. Shen H. M. Hao J. (2024). Macrophage plasticity: signaling pathways, tissue repair, and regeneration. MedComm 5, e658. 10.1002/mco2.658 39092292 PMC11292402

[B37] Yang W. S. Jeong D. Yi Y. S. Park J. G. Seo H. Moh S. H. (2013). IRAK1/4-targeted anti-inflammatory action of caffeic acid. Mediat. Inflamm. 2013, 518183. 10.1155/2013/518183 24379523 PMC3863464

[B38] Ye G. Jimo R. Lu Y. Kong Z. Axi Y. Huang S. (2024). Multifunctional natural microneedles based methacrylated Bletilla striata polysaccharide for repairing chronic wounds with bacterial infections. Int. J. Biol. Macromol. 254, 127914. 10.1016/j.ijbiomac.2023.127914 37939765

[B39] Yue X. Zhao S. Qiu M. Zhang J. Zhong G. Huang C. (2023). Physical dual-network photothermal antibacterial multifunctional hydrogel adhesive for wound healing of drug-resistant bacterial infections synthesized from natural polysaccharides. Carbohydr. Polym. 312, 120831. 10.1016/j.carbpol.2023.120831 37059558

[B40] Zhang X. Liang Y. Huang S. Guo B. (2024). Chitosan-based self-healing hydrogel dressing for wound healing. Adv. Colloid Interface Sci. 332, 103267. 10.1016/j.cis.2024.103267 39121832

[B41] Zhang D. Gao W. Cui X. Qiao R. Li C. (2024). Caffeic acid and cyclen-based hydrogel for synergistic antibacterial therapy. ACS Appl. Mater. Interfaces 16, 44493–44503. 10.1021/acsami.4c09037 39143929

[B42] Zhao P. Guo Z. Wang H. Zhou B. Huang F. Dong S. (2023). A multi-crosslinking strategy of organic and inorganic compound bio-adhesive polysaccharide-based hydrogel for wound hemostasis. Biomater. Adv. 152, 213481. 10.1016/j.bioadv.2023.213481 37307771

[B43] Zhou H. Gao S. J. Zhang M. T. Jia J. Chen F. X. Chen C. L. (2023). Synthesis, configurational analysis and antiviral activities of novel diphenylacrylic acids with caffeic acid as the lead compound. J. Mol. Struct. 1291, 136016. 10.1016/j.molstruc.2023.136016

